# Fibroblasts in Nodular Sclerosing Classical Hodgkin Lymphoma Are Defined by a Specific Phenotype and Protect Tumor Cells from Brentuximab-Vedotin Induced Injury

**DOI:** 10.3390/cancers11111687

**Published:** 2019-10-30

**Authors:** Katrin Bankov, Claudia Döring, Adam Ustaszewski, Maciej Giefing, Marco Herling, Chiara Cencioni, Francesco Spallotta, Carlo Gaetano, Ralf Küppers, Martin-Leo Hansmann, Sylvia Hartmann

**Affiliations:** 1 Dr. Senckenberg Institute of Pathology, Goethe University, 60590 Frankfurt am Main, Germany; katrin.bankov@kgu.de (K.B.); c.doering@em.uni-frankfurt.de (C.D.); martin-leo.hansmann@kgu.de (M.-L.H.); 2 Department of Internal Medicine 1, Hospital of the J.W. Goethe University, 60590 Frankfurt am Main, Germany; 3 Institute of Human Genetics, Polish Academy of Sciences, 60-479 Poznan, Poland; adam.ustaszewski@igcz.poznan.pl (A.U.); giefingm@man.poznan.pl (M.G.); 4 The Laboratory of Lymphocyte Signalling and Oncoproteome, Department I of Internal Medicine, Center for Integrated Oncology (CIO) Aachen-Bonn-Cologne-Duesseldorf, CECAD and CMMC, University of Cologne, 50937 Cologne, Germany; marco.herling@uk-koeln.de; 5 National Research Council (CNR), Institute for Systems Analysis and Computer Science, 00185 Rome, Italy; chcencioni@gmail.com; 6 Department of Oncology, University of Turin, 10060 Candiolo (Turin), Italy; fspallotta@gmail.com; 7 Candiolo Cancer Institute, FPO-IRCCS, 10060 Candiolo (Turin), Italy; 8 Laboratorio di Epigenetica, Istituti Clinici Scientifici Maugeri IRCCS, 27100 Pavia, Italy; carlo.gaetano@icsmaugeri.it; 9 Institute of Cell Biology (Cancer Research), University of Duisburg-Essen, 45122 Essen, Germany; ralf.kueppers@uk-essen.de; 10 Reference and Consultant Center for Lymph Node and Lymphoma Pathology, Goethe University, 60590 Frankfurt am Main, Germany; 11 Frankfurt Institute of Advanced Studies, 60438 Frankfurt am Main, Germany

**Keywords:** classical Hodgkin lymphoma, nodular sclerosis, fibroblasts, gene expression analysis, methylation profiling, luteolin

## Abstract

Classical Hodgkin lymphoma (cHL) is one of the most common malignant lymphomas in Western Europe. The nodular sclerosing subtype of cHL (NS cHL) is characterized by a proliferation of fibroblasts in the tumor microenvironment, leading to fibrotic bands surrounding the lymphoma infiltrate. Several studies have described a crosstalk between the tumour cells of cHL, the Hodgkin- and Reed-Sternberg (HRS) cells, and cancer-associated fibroblasts. However, to date a deep molecular characterization of these fibroblasts is lacking. Thus, the aim of the present study is a comprehensive characterization of these fibroblasts. Gene expression profiling and methylation profiles of fibroblasts isolated from primary lymph node suspensions revealed persistent differences between fibroblasts obtained from NS cHL and lymphadenitis. NS cHL derived fibroblasts exhibit a myofibroblastic phenotype characterized by myocardin (*MYOCD*) expression. Moreover, *TIMP3*, an inhibitor of matrix metalloproteinases, was strongly upregulated in NS cHL fibroblasts, likely contributing to the accumulation of collagen in sclerotic bands of NS cHL. As previously shown for other types of cancer-associated fibroblasts, treatment by luteolin could reverse this fibroblast phenotype and decrease TIMP3 secretion. NS cHL fibroblasts showed enhanced proliferation when they were exposed to soluble factors released from HRS cells. For HRS cells, soluble factors from fibroblasts were not sufficient to protect them from Brentuximab-Vedotin induced cell death. However, HRS cells adherent to fibroblasts were protected from Brentuximab-Vedotin induced injury. In summary, we confirm the importance of fibroblasts for HRS cell survival and identify TIMP3 which probably contributes as a major factor to the typical fibrosis observed in NS cHL.

## 1. Introduction

Classical Hodgkin lymphoma (cHL) is one of the most frequent malignant lymphomas. Unlike in most other types of B cell lymphoma, the tumor cells in HL, the Hodgkin-Reed-Sternberg (HRS) cells only represent a minority in the infiltrate, frequently around 1% [1]. In Western Europe, the nodular sclerosing (NS cHL) subtype is most frequently encountered. This subtype mainly affects adolescents, occurs frequently in mediastinal localization and is usually not Epstein–Barr virus-associated [2]. In this subtype, a proliferation of fibroblasts is observed, leading to sclerosing bands that confine nodular compartments [3].

Fibroblastic reticular cells (FRCs) are a specialized form of myofibroblasts which create the lymph node skeleton with its conduit system [4]. The most important role of FRCs located in the paracortex of lymph nodes is to direct lymphocyte trafficking. Malignant cells recruit and re-educate their surrounding cells to establish a tumor-supportive milieu. This also affects the biology and function of FRCs. Once reprogrammed to cancer-associated fibroblasts (CAFs), they can induce remodeling of the extracellular matrix (ECM) [5]. It was reported that CAFs are able to protect lymphoma cells from apoptosis induced by chemotherapeutic treatments with Rituximab, Bortezomib, or ABT-737 in vitro [6,7,8]. The protective effect seems to be achieved through the induction of drug tolerance and anti-apoptotic signalling [9]. Accordingly, the induction of ABC-transporters was shown in vitro for CAFs isolated from lymph nodes affected by follicular lymphoma and diffuse large B cell lymphoma [9]. In the microenvironment of NS HL, the CAFs and the collagen-rich ECM are engaged in crosstalk with the HRS cells [10,11,12,13,14,15,16]. HRS cells secrete interleukin (IL-)7, which stimulates CAFs to produce high levels of IL-6, acting as a costimulatory factor for regulatory T cells (T_regs_) and helping to create a tumor-supportive microenvironment [15]. Both primary HRS cells and HL-derived cell lines were shown to express IL-6 and IL-7 receptors revealing an important paracrine crosstalk [17,18]. HRS cells attract several types of bystander cells by secretion of CCL5, including eosinophils, mast cells and CD4^+^ T cells [19,20]. Furthermore, they secrete IL-13 [21] and tumor necrosis factor alpha (TNFα), which induce eotaxin and CCL5 expression in fibroblasts and thus attract eosinophils. In NS HL, eosinophils were shown to display high levels of mRNA of tumor growth factor beta (TGFβ)—a potent regulator of proliferation [22]. The production of pro-fibrotic cytokines is higher in NS HL compared to mixed cellularity (MC) HL [23,24], thus explaining the pronounced fibrosis and sclerosis in the NS HL subtype.

At present, literature suggests that CAFs are responsible for the typical fibrosis and sclerosis that is observed in NS HL and are important for the survival of HRS cells. However, the underlying molecular mechanisms are only partly understood. The central aim of the present study, therefore, was to characterize these HL-associated CAFs and elucidate the mechanisms involved in their crosstalk with HRS cells.

## 2. Results 

### 2.1. Fibroblast Cultures Obtained from Primary Lymph Node Suspensions Show a High Purity

Fibroblasts were isolated from cell suspensions of cHL and lymphadenitis lymph node biopsy specimens. To confirm that highly pure fibroblast cultures were obtained from primary suspensions, several steps of quality control were undertaken. The cells obtained from lymph node suspensions after five passages showed the typical fusiform morphology of fibroblasts with protrusions, plastic adherence alongside the expression of the surface markers CD29, CD73, CD90, and CD105 in >94% of the cells (Figure S1). This confirms the high purity of fibroblasts. Expression of CD45, CD146, and CD271 was lacking in the adherent cells, indicating that all cells of lymphoid origin were eliminated through the passaging procedures.

### 2.2. Fibroblasts Derived from NS cHL Differ in Their Gene Expression Patterns from Fibroblasts Isolated from MC cHL or Reactive Lymph Nodes

**Figure 1:**
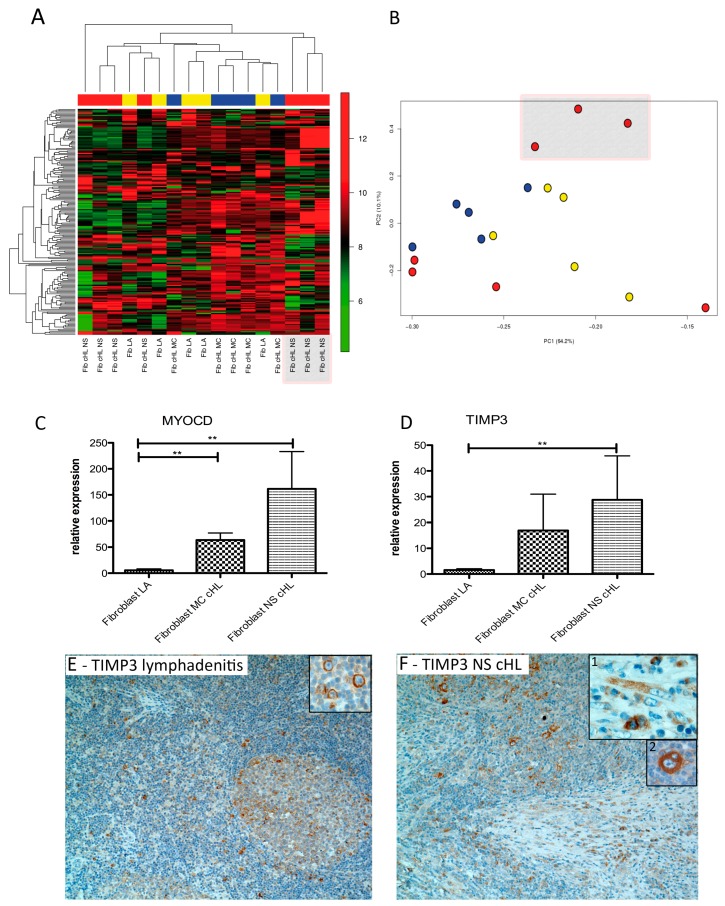
Fibroblasts derived from nodular sclerosing subtype of cHL (NS cHL) and lymphadenitis (LA) show considerable differences in their gene expression program. (**A**) Unsupervised hierarchical gene expression clustering of fibroblast samples derived from lymphadenitis (Fib LA, yellow, *n* = 5), from mixed cellularity subtype of cHL (MC cHL, blue, *n* = 5) and from NS cHL (red, *n* = 7) considering 185 transcripts with a standard deviation >1. (**B**) Principal component analysis considering the same 185 transcripts with a standard deviation >1. Fibroblasts from LA yellow, MC cHL blue) and from NS cHL (red). (**C**) Quantitative real time PCR showing significantly higher myocardin (*MYOCD*) transcript levels in MC cHL (*n* = 5) and NS cHL (*n* = 8) compared with fibroblasts from lymphadenitis (*n* = 5) (Mann–Whitney test, ** *p* < 0.01). (**D**) Quantitative real time PCR showing significantly higher tissue inhibitor of metalloproteinase 3 (*TIMP3*) transcript levels in NS cHL (*n* = 8) compared with fibroblasts from lymphadenitis (*n* = 5) (Mann–Whitney test, ** *p* = 0.002). (**E**) Representative immunohistochemical TIMP3 staining of a lymphadenitis case (100×). TIMP3 is expressed in paraimmunoblasts (insert, 400×). (**F**) Representative immunohistochemical staining for TIMP3 of a NS cHL (100×) with expression of TIMP3 in fibroblasts (insert 1) and Hodgkin- and Reed-Sternberg (HRS) cells (insert 2).

Gene expression profiling using Affymetrix Gene Arrays 1.0 was performed after fibroblasts were kept in culture for five passages and a quality check for fibroblast purity was carried out. Unsupervised hierarchical clustering of all samples (seven NS cHL, five MC cHL, and five lymphadenitis) showed a core group comprising all five fibroblast samples of MC cHL and lymphadenitis as well as three of seven NS cHL cases (Figure 1A). The set of four NS cHL fibroblasts clustered separately from this core group. A similar segregation was observed in the principal component analysis (Figure 1B).

In a supervised comparison between NS cHL fibroblasts and those from lymphadenitis, only one gene turned out to be differentially regulated: *DSC3*, which was downregulated by 4.7-fold in NS cHL fibroblasts (*p* < 0.05 and a false discovery rate (FDR) < 0.3). *DSC3* belongs to the cadherin family and its function is to promote cellular adhesion. It is strongly expressed in fetal mesenchymal stromal cells and downregulated in bone marrow derived stromal cells [25]. The fact that only one gene turned out to be significantly deregulated in this comparison was due to the strong heterogeneity of primary fibroblasts obtained from NS cHL and the relatively small sample size. Considering the genes with *p* < 0.05 and an FDR > 0.3, tissue inhibitor of metalloproteinase 3 (*TIMP3*) and myocardin (*MYOCD*) were most strongly upregulated (6.0- and 3.5-fold) in NS cHL fibroblasts. When all cHL fibroblasts (NS cHL and MC cHL fibroblasts) were compared with lymphadenitis fibroblasts, *TIMP3* and *MYOCD* were most strongly and significantly upregulated (4.2- and 3.9-fold, respectively, filter criteria *p* < 0.05 and FDR < 0.3, Table 1). *TIMP3* is an inhibitor of matrix metalloproteinases and thus contributes to the inhibition of ECM degradation and leads in consequence to the accumulation of ECM [26]. *MYOCD* is a nuclear transcriptional co-activator that plays a crucial role in the differentiation of smooth muscle cell lineage [27]. *DSC3* was again the most strongly downregulated gene (4.0-fold). CXCR4 and SDF1 were not deregulated. In the comparison between NS cHL fibroblasts and MC cHL fibroblasts 14 transcripts were downregulated with a fold change <−2.0 (*p* < 0.05, no filter on FDR, Table S1). Among these, IL-7R was 2.7-fold downregulated in NS cHL fibroblasts, which was previously described to be upregulated in NS cHL fibroblasts in one publication by Cattaruzza et al. [15].

Validation of gene expression by Taqman quantitative real time PCR confirmed a significantly higher expression of *MYOCD* mRNA both in MC cHL and NS cHL fibroblasts when compared with lymphadenitis fibroblasts (Figure 1C, Mann–Whitney test, *p* = 0.008 and *p* = 0.002, respectively). *TIMP3* was expressed at significantly higher levels in NS cHL fibroblasts when compared to lymphadenitis fibroblasts (19-fold, Mann–Whitney test, *p* = 0.002, Figure 1D). Immunohistochemistry was carried out for TIMP3. TIMP3 was not only expressed in the fibroblasts of 7/15 NS cHL cases and 1/11 MC cHL cases, but also in the HRS cells of 14/14 NS cHL and 9/11 MC cHL (Figure 1E,F), implicating that not only fibroblasts contribute to the accumulation of ECM via TIMP3 secretion, but also HRS cells themselves.

### 2.3. Fibroblasts Derived from NS cHL Maintain Stable Methylation Profiles in Culture When Compared with Lymphadenitis-Derived Fibroblasts 

**Figure 2:**
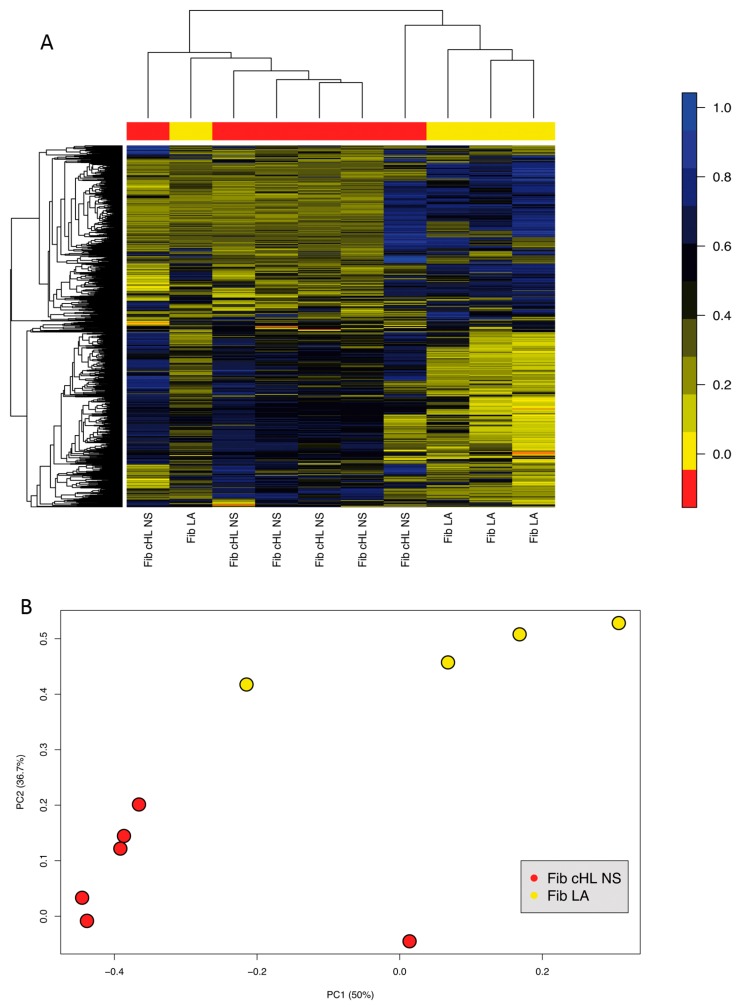
Methylation profiles remain consistent in fibroblasts obtained from lymphadenitis and NS cHL. (**A**) Unsupervised hierarchical clustering of fibroblasts from lymphadenitis (Fib LA, *n* = 4, yellow) and from NS cHL (*n* = 6, red) considering all tags (848) with a differential methylation and standard deviation >0.25. (**B**) Principal component analysis of methylation patterns in fibroblasts from lymphadenitis (Fib LA, *n* = 4, yellow) and NS cHL (*n* = 6, red) considering all tags with a differential methylation and standard deviation >0.25.

Since differences in gene expression between different fibroblast subsets were observed, fibroblasts from six cases of NS cHL and four cases of lymphadenitis obtained after five passages were studied for their methylation profiles using Methylation EPIC BeadChip Kit that interrogates 850,000 CpG sites in the human genome, to reveal if the differences in gene expression are linked to distinct DNA methylation profiles. In an unsupervised hierarchical clustering, fibroblasts from NS cHL and lymphadenitis separated well from each other with the exception of one outlier each (Figure 2A). In a principal component analysis both fibroblasts groups were considerably different (Figure 2B). In the supervised comparison, there were 5815 tags that were significantly differentially methylated (*p* < 0.05 (*p*-values adjusted using an FDR approach)) with mean differences of more than 30% (not shown). A total of 170 tags among these were located in promoter regions. Correlation of gene expression data and methylation results, however, revealed that only for one gene, which was differentially methylated in NS cHL fibroblasts and lymphadenitis (*SLC38A1*; tag: cg17090968 ) a respective significant regulation of gene expression (1.8-fold higher expression in NS cHL fibroblasts, *p* = 0.012 and 45% lower methylation) was observed. Thus, differential expression of genes between these different fibroblast types at their mRNA level is not predominantly regulated by methylation of their gene promoters.

### 2.4. HRS Cells Show a Strong Adherence to Fibroblasts, Which Is Partly Mediated by CD29

**Figure 3:**
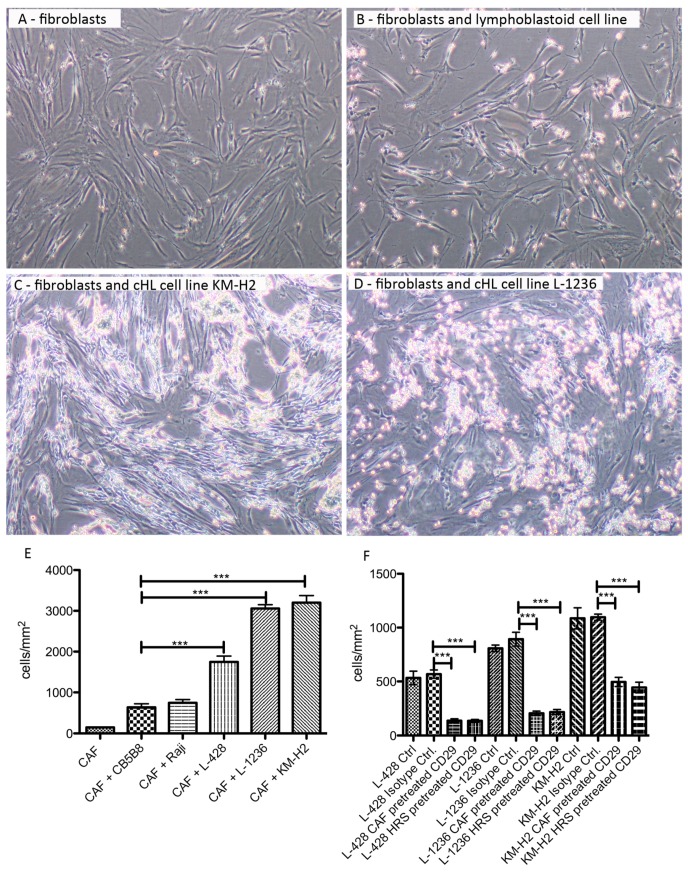
HRS cells from cHL cell lines show strong adherence to NS cHL fibroblasts that can be partly blocked by anti-CD29 antibody. (**A**) Representative image of cancer-associated fibroblast (CAF) monoculture obtained from a NS cHL (4× magnification). (**B**) Representative image of cancer-associated fibroblasts (CAF) cocultured with a lymphoblastoid cell line after removal of the floating cells in suspension by washing of the fibroblasts (4× magnification). (**C**) Representative image of cancer-associated fibroblasts (CAF) cocultured with the cHL cell line KM-H2 after removal of the floating cells in suspension by washing of the fibroblasts (4× magnification). (**D**) Representative image of cancer-associated fibroblasts (CAF) cocultured with the cHL cell line L-1236 after removal of the floating cells in suspension by washing of the fibroblasts (4× magnification). (**E**) Quantification of cells adhering to cancer-associated fibroblasts (CAF) after coculture for 12 h and removal of the floating cells in suspension by washing of the fibroblasts. Means + SEM of four independent experiments in duplicates, *** *p* < 0.001, One-Way-ANOVA with Bonferroni correction for multiple comparisons. (**F**) Pre-incubation of cancer-associated fibroblasts (CAF) or HRS cells with CD29 blocking antibodies before coculture significantly decreases adherence of HRS cells to fibroblasts. Either HRS cells or fibroblasts were pre-incubated with the CD29 blocking antibody. Means + SEM of three or six independent experiments in triplicates, *** *p* < 0.001, One-Way-ANOVA with Bonferroni correction for multiple comparisons.

Assuming that cHL fibroblasts belong to the normal lymph node skeleton and undergo reprogramming by HRS cells, we were interested in, if there is a direct interaction between HRS cells and fibroblasts. The NS cHL cell line L-428, MC cHL cell lines L-1236 and KM-H2 as well as a lymphoblastoid cell line and the Burkitt lymphoma cell line Raji were cocultured with fibroblasts from NS cHL for 12 hours. Whereas all HRS cells—independent of the cHL subtype—showed a firm adherence to fibroblasts, lymphoblastoid and Raji cells could be flushed away more easily by mechanical disruption (Figure 3A–E). Due to the strong adherence of HRS cells to fibroblasts, it was not possible to separate these two fractions for subsequent molecular analysis of the individual populations. Since adhesion molecules play an important role in the adhesion of different cell types, we blocked some of these molecules using antibodies directed against the adhesion molecules CD18, CD29, CD47, CD49, and CD54 in HRS cell and in their fibroblast cocultures. While the number of HRS cells sticking to fibroblasts were not substantially different after application of antibodies directed against CD18, CD47, CD49, and CD54 to the cocultures (data not shown), blocking CD29 resulted in a significant decrease in adhering HRS cells (Figure 3F), indicating that CD29 is an important factor for the direct interaction between HRS cells and fibroblasts.

### 2.5. Conditioned Medium from cHL Cell Lines Has a Growth Promoting Effect on NS cHL Fibroblasts

**Figure 4:**
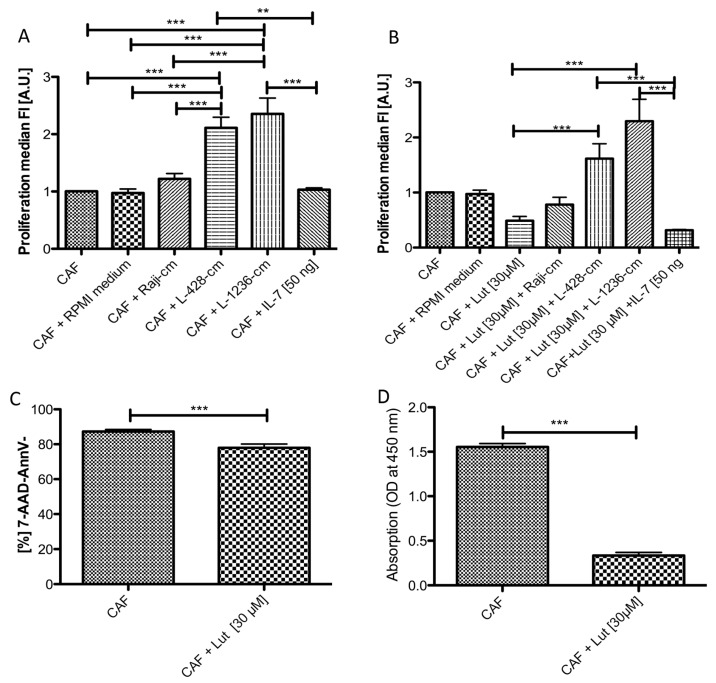
NS cHL fibroblasts show enhanced proliferation in the presence of conditioned medium derived from cHL cell lines, even after application of luteolin. (**A**) NS cHL fibroblasts (CAF) show a significantly higher proliferation after the application of conditioned media from the cHL cell lines L-428 and L-1236 when compared with standard (IMDM) or mock cell culture medium (RPMI 1640 medium) only orconditioned medium obtained from the Burkitt lymphoma cell line Raji or IL-7. Proliferation was measured by Cell Trace Violet Blue. Fluorescence values were inverted and normalized to the control (CAF). Mean + SEM of three independent experiments in triplicates, ** *p* < 0.01, *** *p* < 0.001, One-Way ANOVA with Bonferroni correction for multiple comparisons. (**B**) NS cHL fibroblasts (CAF) show a significantly decreased proliferation after the application of luteolin, which can be reversed by addition of conditioned media from the cHL cell lines L-428 and L-1236, but not by addition of IL-7. Proliferation was measured by Cell Trace Violet Blue. Fluorescence values were inverted and normalized to the control (CAF). Mean + SEM of three independent experiments in triplicates,* *p* < 0.05, Kruskal–Wallis test with Dunn’s correction for multiple comparisons. (**C**) Viable cells in untreated and luteolin treated fibroblasts 48 h after luteolin treatment. *** *p* < 0.001 unpaired *t*-test. Four experiments with three replicates. (**D**) Absorption at 450 nm is significantly decreased in the supernatants derived from luteolin treated fibroblasts after 48 h reflecting a significantly downregulated TIMP3 secretion in the ELISA. Mean + SEM of four independent experiments in triplicates. *** *p* < 0.001, paired *t*-test.

Since we assumed that HRS cells and fibroblasts have a stimulatory or protective effect on each other, we first analysed the effect of soluble factors from cHL cell lines on fibroblasts. It has been described that HRS cells secrete IL-7, which binds to IL-7R on NS cHL fibroblasts [15]. We were therefore interested if IL-7 and conditioned medium from HRS cells have a growth promoting effect on NS cHL fibroblasts. NS cHL fibroblasts showed a significantly higher proliferation when they were cultured with conditioned medium from the cHL cell lines L-428 and L-1236 (Figure 4A). In contrast, no growth promoting effect was observed after the application of 50 ng/mL IL-7 (Figure 4A). We thus conclude that soluble factors secreted from the HRS cells other than IL-7 are responsible for the enhanced growth of fibroblasts.

### 2.6. Luteolin Has a Growth Inhibitory and Reprogramming Effect on NS cHL Fibroblasts, Which Is Abolished by cHL Conditioned Medium

Next, we wanted to clarify if this growth-promoting effect induced by cHL conditioned medium is still active when such fibroblasts were previously treated by luteolin. Luteolin is a flavonoid that has inhibitory effects on IL-6 and TNFα secretion by suppressing NF-κB activity [28]. It has been suggested that luteolin can reverse a myofibroblastic phenotype, which is frequently acquired by fibroblasts during carcinogenesis and which was also observed in NS cHL fibroblasts with respect to the observed overexpression of *MYOCD* [29]. Application of 30µg/mL cell culture volume luteolin reduced the proliferation rate of NS cHL fibroblasts (Figure 4B). Addition of conditioned medium from the cHL cell lines L-428 and L-1236 to luteolin treated NS cHL fibroblasts completely restored proliferation. In contrast, addition of IL-7 to luteolin treated NS cHL could not restore this effect, indicating that factors other than IL-7 in the conditioned medium of cHL cell lines must be responsible for this effect (Figure 4B).

Gene expression analysis of luteolin treated NS cHL fibroblasts using Affymetrix Gene Arrays 1.0 was performed and compared with profiles of untreated NS cHL fibroblasts. Seventy transcripts were ≥2.0-fold upregulated in NS cHL fibroblasts (Table S2) while 125 transcripts were significantly downregulated (Table S3), including several genes that were previously found to be upregulated in cHL fibroblasts, among these *TIMP3* (5.5-fold downregulated) and *MYOCD* (2.0-fold downregulated). These genes defined the NS cHL fibroblast phenotype when compared with lymphadenitis fibroblasts. Hence, we could confirm that luteolin not only inhibits the growth of fibroblasts, but additionally contributes to the downregulation of genes important for the NS cHL fibroblast phenotype. Members of the NF-κB pathway were not significantly downregulated, nor were apoptosis related genes upregulated. Despite the fact that the number of viable cells after luteolin treatment was significantly decreased after 48 hours, the majority of cells that were subjected to gene expression analysis were viable (Figure 4C). Additionally, a significantly decreased secretion of TIMP3 protein in the cell culture supernatants of luteolin treated NS cHL fibroblasts was observed when compared with untreated NS cHL fibroblasts as demonstrated by ELISA (Figure 4D).

### 2.7. HRS Cells Require Direct Fibroblast Contact to Gain Protection against Brentuximab-Vedotin

**Figure 5:**
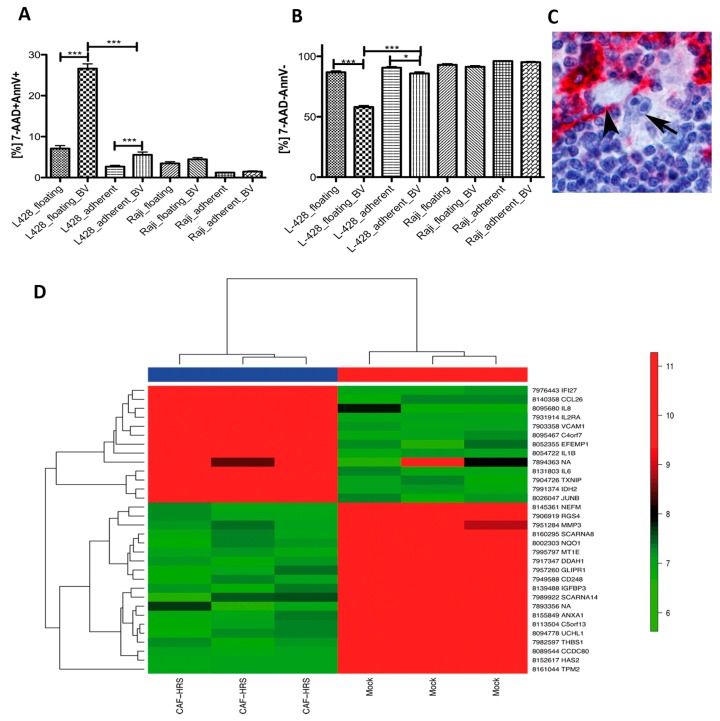
HRS cells adhering to NS cHL fibroblasts are protected from Brentuximab-Vedotin treatment and induce changes in the gene expression program of NS cHL fibroblasts. (**A**) Number of 7-AAD and Annexin V double positive deceased cells are significantly increased in L-428 cells after Brentuximab-Vedotin (BV) treatment. This effect is particularly observed in the cells free floating in suspension and to a lesser degree in L-428 cells adherent to NS cHL fibroblasts. Mean + SEM of three independent experiments in triplicates, *** *p* < 0.001, Mann–Whitney test. (**B**) Number of 7-AAD and Annexin V double negative cells, representing the viable population, are substantially decreased in floating L-428 cells after Brentuximab-Vedotin (BV) treatment. This effect is less dramatic in L-428 cells adherent to NS cHL fibroblasts. Mean + SEM of three independent experiments in triplicates, *** *p* < 0.001, Mann–Whitney test. (**C**) Example of a primary HRS cell (arrow) adhering to a CD10-positive myofibroblastic cell (arrow head) in a case of primary cHL NS. CD10-immunostaining, 400× magnification. (**D**) Unsupervised hierarchical clustering of gene expression profiles derived from NS cHL fibroblasts (CAF) and HRS cells of the cHL cell line L-428 after 48 h coculture and mixture of NS cHL fibroblasts (CAF) and HRS cells of the cHL cell line L-428 in the same ratio immediately prior to gene expression profiling (Mock). The cluster considers all genes with a standard deviation ≥1 which was observed in 32 genes.

It is commonly known that the presence of stroma cells like fibroblasts can provide a pro-survival advantage to cancer cells [30]. Here we were interested to clarify whether HRS cells need a direct interaction with fibroblasts or whether soluble factors delivered from fibroblasts would be sufficient to provide this effect. First, HRS cells of the cHL cell lines L-428 and L-1236 were incubated with supernatants from fibroblasts. However, no effect on viability or proliferation was observed (data not shown). Next, L-428 cells were cocultured with NS cHL fibroblasts in a ratio of 5:1 for 12 h. In this setting, 50 µg/mL cell culture volume Brentuximab-Vedotin, a CD30-specific antibody drug conjugate, was applied as previously published [31]. Cells were stained for Annexin V and 7-amino-actinomycin D (7-AAD) and the proportion of positive cells was determined by flow cytometry after 48 h. For comparison, the same procedure was performed with the CD30-negative Burkitt lymphoma cell line Raji. The number of Annexin V- and 7-AAD-double positive L-428 cells was significantly increased after Brentuximab-Vedotin both in the floating L-428 fraction and in adherent cells, indicating the efficiency of Brentuximab-Vedotin to kill HRS cells (Figure 5A). With respect to the viable cells, only a small, albeit significant reduction in viable cells was observed in the HRS cells adhering to fibroblasts (Figure 5B), indicating that a relevant number of HRS cells can overcome Brentuximab-Vedotin induced reduction of viability when adherent to fibroblasts (Figure 5C). No effect was observed after Brentuximab-Vedotin application to CD30-negative Raji cells.

### 2.8. Coculture of NS cHL Fibroblasts with HRS Cells Induces an Inflammatory Response and a Follicular Dendritic Cell-Like Phenotype

NS cHL fibroblasts were cocultured with L-428 cells in a ratio of 1:5 for 48 h. Cells were harvested and total RNA from the coculture was extracted for subsequent gene expression profiling. Due to the strong adherence of HRS cells to NS cHL fibroblasts, it was not possible to separate these two fractions for independent gene expression profiling as originally planned. Therefore, NS cHL fibroblasts mixed with L-428 cells at the same ratio immediately before RNA extraction served as a control, in order to determine which genes are up- and downregulated during the coculture process. L-428 cells that were previously cocultured with NS cHL fibroblasts showed substantial differences in their gene expression when compared to L-428 cells freshly mixed with NS cHL fibroblasts (Figure 5D). We could rule out that these differences in gene expression might be related to an enhanced proliferation of HRS cells after 48 h when compared with fibroblasts, since *TNFRSF8* (CD30) was not expressed at higher levels after 48 hours of coculture as compared to freshly mixed cells. A total of 61 transcripts was significantly upregulated during this coculture process (Table S4) using cut-off fold change (FC) of ≥2, among these were *IL-1B* (9.1-fold), *CCL26* (5.4-fold), and *FDCSP* (5.2-fold) most strongly upregulated. FDCSP is specifically expressed in follicular dendritic cells [32]. Furthermore, *IL-2RA* (4.4-fold), *JUNB* (4.0-fold), *IL-6* and *IDH2* (both 3.6-fold), as well as *VCAM1* (3.5-fold, involved in cell adhesion), which is upregulated in follicular dendritic cells after contact with lymphocytes [33], were significantly upregulated.

## 3. Discussion

### 3.1. cHL Fibroblasts Differ from Lymphadenitis-Derived Fibroblasts

The aim of this study was to characterize NS cHL fibroblasts. To our knowledge, this is the first study analysing NS cHL fibroblasts obtained from primary lymphoma tissue on a molecular basis. We could confirm a high purity in all samples, being a necessary requisite for all subsequent analyses. Although all fibroblasts were kept in culture up to several weeks in order to accomplish five passages and to obtain highly pure cultures, they still displayed differences in gene expression even when bystander cells were absent. In line with this observation of a conserved gene expression programme, a highly stable methylation pattern was observed between NS cHL fibroblasts and fibroblasts obtained from lymphadenitis. Surprisingly, there was only a very weak correlation between methylation and gene expression, indicating that most genes upregulated in NS cHL fibroblasts after activation by HRS cells are regulated by other mechanisms. In our gene expression analysis, we could confirm previous data showing that IL-6 is an important factor in the crosstalk between NS cHL fibroblasts and HRS cells [10].

### 3.2. TIMP3 and MYOCD Are Important for cHL Fibroblasts

We identified *MYOCD* and *TIMP3* as generally deregulated genes in cHL fibroblasts. Whereas *MYOCD* was expressed at similar levels in NS and MC cHL fibroblasts in the gene expression arrays, *TIMP3* was more strongly expressed in NS cHL fibroblasts. This fits well with the specific accumulation of ECM in the NS cHL type. *MYOCD* is an important transcription factor for the differentiation to myofibroblasts and smooth muscle cells, enhancing transcription of myosin heavy chain *MYH11*, *ACTA2*, *ACTB*, and *ACTG1*, thus promoting contractility of the cells [27,34]. *CNN1*, which is another MYOCD downstream target [27], was also strongly expressed in NS cHL fibroblasts. *MYOCD* overexpression alone was sufficient to induce a mature smooth muscle cell-like phenotype in human embryonic stem cells [34]. Furthermore, MYOCD can lead to increased type I collagen expression [35]. Enhanced expression of *MYOCD* together with *TIMP3*, which inhibits the degradation of ECM are thus likely the factors that are most responsible for the accumulation of fibrotic tissue in the NS cHL subtype. Surprisingly, TIMP3 protein was not only expressed by fibroblasts, but also by the HRS cells of most cHL cases, suggesting that HRS cells themselves also inhibit degradation of ECM and thus contribute to the accumulation of ECM. This is surprising, since *TIMP3* expression is frequently lost in several types of advanced cancers [36,37,38]. Moreover, deletion of the four TIMPs in dermal fibroblasts resulted in a CAF-like phenotype [39]. However, since we here found overexpression of *TIMP3* in NS cHL derived fibroblasts, this cell type apparently differs from CAFs derived from solid cancers. The NS cHL fibroblast phenotype which is acquired by these specialised fibroblasts can be inhibited by luteolin treatment, which also inhibits proliferation and TIMP3 secretion of these cells. Luteolin is an agent, which acts on many different pathways and which cannot be applied as a therapeutic agent. It has been described that IL-7 secreted from HRS cells strongly induced proliferation of fibroblasts [15], however, we could not confirm this observation when adding purified IL-7 to the culture. Thus interfering with IL-6 and IL-7 signalling does not seem to be a promising approach with respect to our data.

### 3.3. cHL Cell Lines and Fibroblasts Strongly Influence Each Other and Deliver Prosurvival Signals

In contrast, conditioned media from cHL cell lines contained the required factors to reverse the effect of luteolin and could even enhance the proliferation of these specialised fibroblasts. We thus conclude that other factors, possible microvesicles as previously described [40], could be responsible for the induction of proliferation. For the proliferation of NS cHL fibroblasts, the presence of soluble factors derived from HRS cells in the medium was fully sufficient. HRS cells cocultured with fibroblasts, in contrast, were protected from Brentuximab-Vedotin induced cell death only, when they were adherent to fibroblasts and thus had direct contact to the stromal cell component. A close contact of HRS cells to surrounding fibroblasts may thus not only be a side effect of increased TIMP3 secretion, but may effectively result in the creation of a niche that is protective for the HRS cells. Induction of VCAM-1 after coculture of fibroblasts and HRS cells as well as the importance of its receptor CD29 in the adhesion between HRS cells and fibroblasts underscores the close interplay relying on cellular adhesion between HRS cells and fibroblasts. Similar observations have been made for bone marrow derived stroma cells which have a proactive role as signalling enhancers for chronic lymphocytic leukemia tumor cells. In the era of targeted therapies, which selectively inhibit the growth of the tumor cells, the supportive and proactive role of the tumor surrounding fibroblasts should not be underestimated [41]. There are first approaches using genetically modified autologous mesenchymal stroma cells delivering tumor killing factors in patients [42], however, this has to be further developed before it can be applied in cHL since patients already have a very high rate of cure using standard regimens. Since conventional combined modality treatment also acts on accompanying fibroblasts it has some effects that are not delivered by targeted therapies and might thus be matter of choice in selected patients. 

## 4. Methods

### 4.1. Fibroblast Isolation from Primary Lymph Nodes

Suspensions of lymph node specimens of five lymphadenitis cases, seven NS cHL and five MC cHL were obtained at the Dr. Senckenberg Institute of Pathology after the diagnosis was established. Tissue samples used in this study were provided by the University Cancer Center Frankfurt (UCT). Written informed consent was obtained from all patients in accordance with the Declaration of Helsinki and the study was approved by the institutional review boards of the UCT and the Ethics Committee at the University Hospital Frankfurt (project-number: SHN-03-2018). Primary suspensions were taken into primary culture for five passages using Iscove’s Modified Dulbecco’s Medium (IMDM) supplemented with 20% fetal calf serum, 1% penicillin-streptomycin (Thermo Fisher Scientific, Waltham, MA, USA) and beta fibroblast growth factor (10 ng/mL, Anaspec, Fremont, CA, USA). The purity of fibroblast cultures was confirmed using the minimal defining criteria of mesenchymal cells [43] by the typical fusiform morphology with protrusions, plastic adherence under culture conditions and the expression of the surface markers CD29, CD73, CD90, and CD105 as well as absence of CD45, CD146, CD271, and CD362 as determined by flow cytometry (Figure S1). After 6 weeks, fibroblasts were harvested and RNA and DNA was extracted (Qiagen DNA Mini Kit, Qiagen RNeasy Mini Kit, Qiagen, Hilden, Germany).

### 4.2. Gene Expression Profiling and Methylation Arrays

Gene expression analysis was performed as previously described [44]. Briefly, RNA was extracted using the RNeasy Mini Kit (Qiagen), cDNA was generated and amplified with the NUGEN Ovation Pico WTA System V2 (NuGEN Technologies, Redwood City, CA, USA) and hybridized onto Affymetrix Gene arrays 1.0 (Affymetrix, Santa Clara, CA, USA), which have probe sets for 28,869 human genes with 764,885 distinct probes. Gene expression data will be available through the GEO database. Unsupervised hierarchical clustering was performed for these genes with a standard deviation ≥1 across all samples using the Manhattan distance and the average linkage method. Gene set characterization analysis was performed using the Genomatix Pathway System (Genomatix, Munich, Germany) at default parameters, listing all canonical pathways and biological terms with a significant enrichment of the provided input genes. Selected targets were validated using quantitative PCR and immunohistochemistry as previously described ([44]; see Supplementary Materials). Furthermore, TIMP3 secretion was analyzed using Human TIMP3 PicoKine ELISA Kit according to manufacturer´s instruction (BosterBio, Pleasanton, CA, USA). 

Methylation profiling was performed using MethylationEPIC BeadChip Kit covering 850,000 CpG sites (Illumina, San Diego, CA, USA) according to the standard protocol. Raw data have been normalized using minfi package [45]. Then data were filtered for promoter tags only. Tags were considered to be differentially methylated, when there was a difference in mean methylation >30% between the two groups and the *p*-value was *p* < 0.05.

### 4.3. Coculture Experiments 

#### 4.3.1. Functional Coculture Experiments

The cHL cell lines L-428 (RRID:CVCL_1361), L-1236 (RRID:CVCL_2096) and KM-H2 (RRID:CVCL_1330), the Burkitt lymphoma cell line Raji (RRID:CVCL_0511) and adherent activated fibroblast cells from a primary NS cHL were used. Due to the limited growth of primary fibroblasts, these functional experiments could not be performed with MC cHL- or lymphadenitis-derived fibroblasts. All cell lines were obtained from the German Collection of Microorganisms and Cell Cultures (DSMZ) and authenticated by STR profiling. A lymphoblastoid cell line CB5B8 used was established from a tonsillar suspension. All experiments were performed with mycoplasma-free cells. The adherent fraction was seeded first to obtain a typical fusiform morphology before lymphoma cells were added. The ratio of adherent primary activated fibroblasts to lymphoma cells was 1:5. Interaction of lymphoma cells and fibroblasts was determined after 12 h of coculture after removing HRS cells in suspension by repeated gentle washing with DPBS (Mg^2+^, Ca^2+^) (100 mg/L each as indicated by manufacturer; Thermo Fisher Scientific, Waltham, MA, USA). Images of the remaining cells adherent to fibroblasts were taken with a brightfield inverted microscope (Olympus CKX41, C-Mount Phototubus U-TV1X-2/U-CMAD3, cellSens Entry v1.11, Tokio, Japan) and the interacting cHL cells were identified and quantified with imageJ (Rasband, W.S., ImageJ, U.S. National Institutes of Health, Bethesda, MD, USA, https://imagej.nih.gov/ij/, 1997–2018, see Supplementary Materials). Details on blocking antibodies for coculture experiments are found in the Supplementary Materials.

#### 4.3.2. Proliferation Assay after Stimulation of Primary Adherent cHL-Derived Fibroblasts and Luteolin Treatment

Primary adherent cHL-derived fibroblasts were supplemented with either lymphoma cell line conditioned supernatant, 30 µM luteolin, IL-7 (Peprotech, Hamburg, Germany) or combined treatments. The proliferation rate was measured using the CellTrace Violet Cell Proliferation Kit (Thermo Fisher Scientific) according to manufacturer´s instruction 48 h after treatment. For each approach the median violet fluorescence intensity was measured, inverted and normalized to the control.

#### 4.3.3. Apoptosis Assay and Treatment with Brentuximab Vedotin

Brentuximab-Vedotin was applied in a dosage of 50 µg/mL as described before [31] and apoptosis rates were determined in both the interacting adherent coculture fraction as well as the non-interacting floating fraction of suspension cells by APC-Annexin V Apoptosis Detection Kit (Thermo Fisher Scientific) including Annexin V and 7-Aminoactinomycin (7-AAD) staining at 48 h. Apoptosis rates of single cHL cell line culture supplemented with fibroblast conditioned media supernatant were also determined after identical Brentuximab-Vedotin treatment. Since L-1236 cells show some ability to adhere on their own to cell culture dishes and KM-H2 HRS cells express CD30 to a lesser extent they have not been selected for this experimental set-up.

### 4.4. Statistical Analysis

All data were tested for the presence of a Gaussian distribution by a Kolmogorov-Smirnov-test. If a Gaussian distribution was present, a One-way-ANOVA test with Bonferroni correction for multiple comparisons or a two-tailed unpaired *t*-test was applied, otherwise as non-parametric tests, a Kruskal-Wallis-test with Dunn´s correction for multiple comparisons or a Mann–Whitney test was performed.

## 5. Conclusions

cHL-derived fibroblasts differ in their gene expression signature as well as methylation from lymphadenitis-derived fibroblasts and are thus reprogrammed by the influence of HRS cells. TIMP3 is particularly expressed in NS cHL fibroblasts and probably contributes to the accumulation of extracellular matrix in this cHL subtype. In contrast, MYOCD, indicating smooth muscle differentiation of fibroblasts, is upregulated both in NS and MC cHL fibroblasts. HRS cells show a direct interaction with cHL-derived fibroblasts via CD29. Attachment of HRS cells to fibroblasts can attenuate Brentuximab-Vedotin induced cell death, suggesting an important role of fibroblasts in the modulation of targeted therapies.

## Figures and Tables

**Table 1:** Genes differentially expressed between fibroblasts derived from cHL and lymphadenitis.

Fold Change Fibroblasts cHL/LA	*p*-Value	False Fiscovery Rate (FDR)	Gene Symbol	Gene Description
4.2	0.006	0.281	TIMP3	TIMP metallopeptidase inhibitor 3
3.9	0.00006	0.138	MYOCD	myocardin
2.0	0.004	0.278	RGS4	regulator of G-protein signalling 4
1.9	0.006	0.281	IER3	immediate early response 3
1.7	0.001	0.187	ENO2	enolase 2 (gamma, neuronal)
1.6	0.005	0.278	SERPINE1	serpin peptidase inhibitor, clade E (nexin, plasminogen activator inhibitor type 1), member 1
1.6	0.004	0.278	PMS2L2	postmeiotic segregation increased 2-like 2 pseudogene
1.6	0.002	0.215	GPX7	glutathione peroxidase 7
1.5	0.005	0.278	CT47A10	cancer/testis antigen family 47, member A10
−1.5	0.004	0.278	TSHZ2	teashirt zinc finger homeobox 2
−1.5	0.0004	0.152	KIAA1598	KIAA1598
−1.6	0.005	0.278	BMP2K	BMP2 inducible kinase
−1.6	0.004	0.278	CD14	CD14 molecule
−1.6	0.006	0.281	OSBPL8	oxysterol binding protein-like 8
−1.6	0.0002	0.138	PDE4DIP	phosphodiesterase 4D interacting protein
−1.7	0.004	0.278	CCNL1	cyclin L1
−1.7	0.004	0.278	SMG1	SMG1 phosphatidylinositol 3-kinase-related kinase
−1.7	0.002	0.215	MOCOS	molybdenum cofactor sulfurase
−1.7	0.005	0.281	SMG1	SMG1 phosphatidylinositol 3-kinase-related kinase
−1.7	0.0002	0.138	RNA5SP187	RNA, 5S ribosomal pseudogene 187
−1.8	0.005	0.281	DSEL	dermatan sulfate epimerase-like
−1.8	0.002	0.230	SCARNA9	small Cajal body-specific RNA 9
−1.8	0.004	0.278	ALPK2	alpha-kinase 2
−1.9	0.005	0.281	TFAP2A	transcription factor AP-2 alpha (activating enhancer binding protein 2 alpha)
−2.0	0.001	0.206	RNA5SP129	RNA, 5S ribosomal pseudogene 129
−2.1	0.003	0.247	PRKG2	protein kinase, cGMP-dependent, type II
−2.2	0.0002	0.138	VIT	vitrin
−2.4	0.0001	0.138	MT-TA	mitochondrially encoded tRNA alanine
−2.7	0.0003	0.138	GPNMB	glycoprotein (transmembrane) nmb
−2.9	0.002	0.215	HTR2B	5-hydroxytryptamine (serotonin) receptor 2B, G protein-coupled
−4.0	0.001	0.161	DSC3	desmocollin 3

## Supplementary Materials

The following are available online at https://www.mdpi.com/2072-6694/11/11/1687/s1, Figure S1: The expression of the surface markers CD29, CD73, CD90, and CD105 as well as absence of CD45, CD146, CD271, and CD362 as determined by flow cytometry, Table S1: Genes downregulated in NS cHL-derived fibroblasts compared with MC cHL-derived fibroblasts, Table S2: Genes upregulated after luteolin treatment in NS cHL-derived fibroblasts compared with untreated NS cHL-derived fibroblasts, Table S3: Genes downregulated after luteolin treatment in NS cHL-derived fibroblasts compared with untreated NS cHL-derived fibroblasts, Table S4: Genes upregulated after 48 h coculture of L-428 cells with NS cHL-derived fibroblasts compared with freshly mixed L-428 cells and NS cHL-derived fibroblasts.
