# Supplementary material for: Fibroblasts in Nodular Sclerosing Classical Hodgkin Lymphoma Are Defined by a Specific Phenotype and Protect Tumor Cells from Brentuximab-Vedotin Induced Injury
Source: Cancers (Basel). 2019 Oct 30;11(11):1687. doi: 10.3390/cancers11111687 (PMC6896072; doi:10.3390/cancers11111687)
Supplement: Supplementary file 1 [file cancers-11-01687-s001.pdf]

cancers

Supplementary Materials

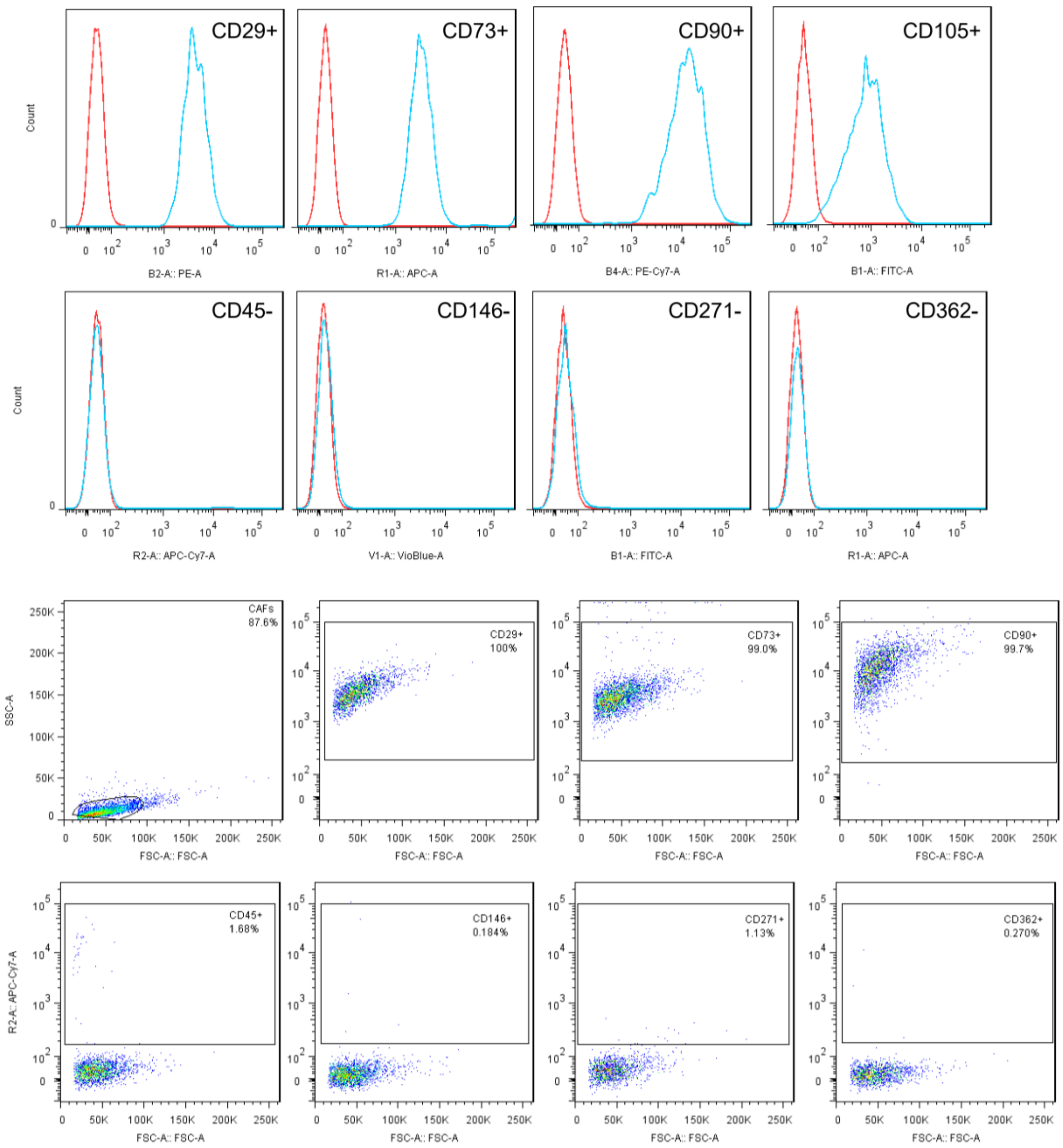

Figure S1. The expression of the surface markers CD29, CD73, CD90, and CD105 as well as absence of CD45, CD146, CD271, and CD362 as determined by flow cytometry (red histogram marks isotype control staining).

Table S1. Genes downregulated in NS cHL-derived fibroblasts compared with MC cHL-derived fibroblasts.

Fold Change Fibroblasts NS/MC cHL	p-value	False discovery rate (FDR)	Gene Symbol	Gene Description
-2.2	0.013	0.979	LOXL4	lysyl oxidase-like 4
-2.2	0.002	0.531	ADM	adrenomedullin
-3.7	0.003	0.638	KRTAP1-5	keratin associated protein 1-5
-2.0	0.003	0.638	SLC14A1	solute carrier family 14 (urea transporter), member 1 (Kidd blood group)
-2.5	0.004	0.638	PSG7	pregnancy specific beta-1-glycoprotein 7 (gene/pseudogene)
-2.7	0.001	0.531	PSG5	pregnancy specific beta-1-glycoprotein 5
-4.7	0.020	0.979	PSG4	pregnancy specific beta-1-glycoprotein 4
-2.7	0.003	0.638	GALNT5	polypeptide N-acetylgalactosaminyltransferase 5
-2.1	0.015	0.979	PRSS12	protease, serine, 12 (neurotrypsin, motopsin)
-2.7	0.018	0.979	IL7R	interleukin 7 receptor
-2.2	0.049	0.979	FBN2	fibrillin 2
-2.1	0.011	0.979	ENPP1	ectonucleotide pyrophosphatase/phosphodiesterase 1
-2.6	0.012	0.979	MOXD1	monooxygenase, DBH-like 1
-2.5	0.032	0.979	CITED2	Cbp/p300-interacting transactivator, with Glu/Asp-rich carboxy-terminal domain, 2

Table S2. Genes upregulated after luteolin treatment in NS cHL-derived fibroblasts compared with untreated NS cHL-derived fibroblasts.

Fold Change Fibroblasts luteolin treated/untreated	p-value	False discovery rate (FDR)	Gene Symbol	Gene Description
4.2	0.003	0.027	SCD	stearoyl-CoA desaturase (delta-9-desaturase)
3.5	0.048	0.095	HMOX1	heme oxygenase (decycling) 1
3.3	0.019	0.054	ID3	inhibitor of DNA binding 3, dominant negative helix-loop-helix protein
3.3	0.000	0.014	TP53INP1	tumor protein p53 inducible nuclear protein 1
3.2	0.000	0.014	BTG2	BTG family, member 2
3.2	0.041	0.085	BDNF	brain-derived neurotrophic factor
3.2	0.003	0.027	TNFRSF10D	tumor necrosis factor receptor superfamily, member 10d, decoy with truncated death domain
3.1	0.003	0.027	FADS1	fatty acid desaturase 1
3.0	0.006	0.033	CTSK	cathepsin K
3.0	0.000	0.014	SESN1	sestrin 1
3.0	0.003	0.027	CDKN1A	cyclin-dependent kinase inhibitor 1A (p21, Cip1)
2.9	0.019	0.053	MIR21	microRNA 21
2.9	0.001	0.019	GDF15	growth differentiation factor 15
2.9	0.002	0.027	MEGF8	multiple EGF-like-domains 8
2.9	0.001	0.019	PPP1R3C	protein phosphatase 1, regulatory subunit 3C

2.8	0.010	0.039	SNAI2	snail family zinc finger 2
2.8	0.006	0.032	SNORD50A	small nucleolar RNA, C/D box 50A
2.8	0.021	0.057	NAMPT	nicotinamide phosphoribosyltransferase
2.8	0.036	0.078	OXTR	oxytocin receptor
2.7	0.004	0.029	HIST1H4I // HIST1H4A // HIST1H4D // HIST1H4F // HIST1H4K // HIST1H4J // HIST1H4C // HIST1H4H // HIST1H4B // HIST1H4E // HIST1H4L // HIST2H4A // HIST4H4 // HIST2H4B	histone cluster 1, H4i // histone cluster 1, H4a // histone cluster 1, H4d // histone cluster 1, H4f // histone cluster 1, H4k // histone cluster 1, H4j // histone cluster 1, H4c // histone cluster 1, H4h // histone cluster 1, H4b // histone clus
2.7	0.007	0.034	ABHD4	abhydrolase domain containing 4
2.6	0.025	0.062	SNORD113-4	small nucleolar RNA, C/D box 113-4
2.6	0.017	0.050	ADM	adrenomedullin
2.6	0.007	0.034	DNER	delta/notch-like EGF repeat containing
2.6	0.000	0.014	ALDOC	aldolase C, fructose-bisphosphate
2.6	0.018	0.053	CITED2	Cbp/p300-interacting transactivator, with Glu/Asp-rich carboxy-terminal domain, 2
2.6	0.003	0.027	EMC3	ER membrane protein complex subunit 3
2.5	0.000	0.017	TMEM45A	transmembrane protein 45A
2.5	0.003	0.027	RABL2B	RAB, member of RAS oncogene family-like 2B
2.5	0.004	0.029	SNORD50B	small nucleolar RNA, C/D box 50B
2.4	0.024	0.061	TSPAN6	tetraspanin 6
2.4	0.001	0.019	ZMAT3	zinc finger, matrin-type 3
2.4	0.006	0.033	ENPP1	ectonucleotide pyrophosphatase/phosphodiesterase 1
2.4	0.021	0.057	LMAN2	lectin, mannose-binding 2
2.4	0.025	0.063	ID1	inhibitor of DNA binding 1, dominant negative helix-loop-helix protein
2.4	0.017	0.050	SDF2	stromal cell-derived factor 2
2.4	0.046	0.092	INHBA	inhibin, beta A
2.3	0.004	0.029	P4HA2	prolyl 4-hydroxylase, alpha polypeptide II
2.3	0.007	0.034	EPB41L4A-AS1	EPB41L4A antisense RNA 1
2.3	0.010	0.039	SNORD104	small nucleolar RNA, C/D box 104
2.3	0.020	0.055	FBXO32	F-box protein 32
2.3	0.005	0.031	TXNRD1	thioredoxin reductase 1
2.3	0.031	0.072	PDGFC	platelet derived growth factor C
2.3	0.002	0.022	TMEM208	transmembrane protein 208
2.3	0.004	0.029	RRM2B	ribonucleotide reductase M2 B (TP53 inducible)
2.3	0.002	0.022	DSEL	dermatan sulfate epimerase-like

2.3	0.000	0.016	TP53TG1	TP53 target 1 (non-protein coding)
2.2	0.009	0.037	NDNF	neuron-derived neurotrophic factor
2.2	0.011	0.042	SNORD3B-1	small nucleolar RNA, C/D box 3B-1
2.2	0.009	0.036	TIMM8B	translocase of inner mitochondrial membrane 8 homolog B (yeast)
2.2	0.001	0.019	C6orf52	chromosome 6 open reading frame 52
2.1	0.039	0.083	WNT5A	wingless-type MMTV integration site family, member 5A
2.1	0.005	0.032	RPL13	ribosomal protein L13
2.1	0.003	0.027	ERGIC3	ERGIC and golgi 3
2.1	0.000	0.018	MYL5	myosin, light chain 5, regulatory
2.1	0.005	0.032	RAB13	RAB13, member RAS oncogene family
2.1	0.000	0.014	TRIM22	tripartite motif containing 22
2.1	0.021	0.056	SNORA61	small nucleolar RNA, H/ACA box 61
2.1	0.008	0.035	PTCH2	patched 2
2.1	0.008	0.036	CYB5D2	cytochrome b5 domain containing 2
2.1	0.010	0.039	GRN	granulin
2.1	0.003	0.027	ATP10D	ATPase, class V, type 10D
2.1	0.001	0.019	YBEY	ybeY metallopeptidase (putative)
2.1	0.004	0.029	RNU1-106P	RNA, U1 small nuclear 106, pseudogene
2.0	0.001	0.019	GPX4	glutathione peroxidase 4
2.0	0.006	0.033	TM9SF4	transmembrane 9 superfamily protein member 4
2.0	0.007	0.034	EIF4B	eukaryotic translation initiation factor 4B
2.0	0.004	0.029	PIGS	phosphatidylinositol glycan anchor biosynthesis, class S
2.0	0.012	0.042	HTRA1	HtrA serine peptidase 1
2.0	0.014	0.046	COX6A1	cytochrome c oxidase subunit VIa polypeptide 1

Table S3. Genes downregulated after luteolin treatment in NS cHL-derived fibroblasts compared with untreated NS cHL-derived fibroblasts.

Fold Change Fibroblasts luteolin treated/untreated	p-value	False discovery rate (FDR)	Gene Symbol	Gene Description
-12.2	0.007	0.035	BIRC5	baculoviral IAP repeat containing 5
-11.6	0.001	0.022	HIST1H3B	histone cluster 1, H3b
-7.9	0.021	0.057	TOP2A	topoisomerase (DNA) II alpha 170kDa
-7.7	0.016	0.049	ANLN	anillin, actin binding protein
-6.8	0.007	0.034	PRR11	proline rich 11
-6.6	0.030	0.069	CENPF	centromere protein F, 350/400kDa
-5.9	0.039	0.082	TPX2	TPX2, microtubule-associated
-5.8	0.017	0.051	PRC1	protein regulator of cytokinesis 1
-5.8	0.004	0.029	CCNB1	cyclin B1
-5.5	0.003	0.029	**TIMP3**	TIMP metallopeptidase inhibitor 3
-5.2	0.025	0.063	ASPM	asp (abnormal spindle) homolog, microcephaly associated (Drosophila)
-5.2	0.011	0.042	PTTG1	pituitary tumor-transforming 1
-5.1	0.019	0.054	MKI67	marker of proliferation Ki-67
-5.0	0.004	0.029	RRM2	ribonucleotide reductase M2

-4.8	0.024	0.062	ESM1	endothelial cell-specific molecule 1
-4.8	0.033	0.075	PLK1	polo-like kinase 1
-4.8	0.001	0.021	**TIMP3**	TIMP metallopeptidase inhibitor 3
-4.6	0.015	0.048	NCAPG	non-SMC condensin I complex, subunit G
-4.6	0.008	0.035	UBE2T	ubiquitin-conjugating enzyme E2T (putative)
-4.5	0.037	0.080	NUSAP1	nucleolar and spindle associated protein 1
-4.4	0.011	0.041	CCL2	chemokine (C-C motif) ligand 2
-4.4	0.006	0.032	UBE2C	ubiquitin-conjugating enzyme E2C
-4.3	0.018	0.052	CENPW	centromere protein W
-4.3	0.025	0.062	CCNB2	cyclin B2
-4.3	0.023	0.060	CCNA2	cyclin A2
-4.2	0.025	0.063	CASC5	cancer susceptibility candidate 5
-4.2	0.025	0.062	CENPE	centromere protein E, 312kDa
-4.0	0.001	0.020	OCIAD2	OCIA domain containing 2
-4.0	0.018	0.052	**IL6**	interleukin 6
-3.9	0.016	0.050	AURKA	aurora kinase A
-3.9	0.006	0.032	**NTN4**	netrin 4
-3.8	0.016	0.049	HIST1H2BM	histone cluster 1, H2bm
-3.8	0.019	0.053	KIF23	kinesin family member 23
-3.8	0.019	0.053	BIRC5	baculoviral IAP repeat containing 5
-3.8	0.020	0.054	**LTBP1**	latent transforming growth factor beta binding protein 1
-3.8	0.012	0.042	**KRT19**	keratin 19
-3.7	0.023	0.060	CYP1B1	cytochrome P450, family 1, subfamily B, polypeptide 1
-3.7	0.013	0.044	KPNA2	karyopherin alpha 2 (RAG cohort 1, importin alpha 1)
-3.6	0.017	0.051	CDKN3	cyclin-dependent kinase inhibitor 3
-3.6	0.031	0.071	BUB1	BUB1 mitotic checkpoint serine/threonine kinase
-3.5	0.023	0.060	NFIB	nuclear factor I/B
-3.4	0.011	0.040	CDC20	cell division cycle 20
-3.4	0.004	0.029	STMN1	stathmin 1
-3.4	0.032	0.073	KIF4A	kinesin family member 4A
-3.3	0.043	0.089	KIF11	kinesin family member 11
-3.2	0.034	0.076	HJURP	Holliday junction recognition protein
-3.1	0.002	0.023	FABP5	fatty acid binding protein 5 (psoriasis-associated)
-3.1	0.011	0.041	MLLT11	myeloid/lymphoid or mixed-lineage leukemia (trithorax homolog, Drosophila); translocated to, 11
-3.1	0.013	0.044	TACC3	transforming, acidic coiled-coil containing protein 3
-3.1	0.033	0.075	CKAP2L	cytoskeleton associated protein 2-like
-3.1	0.020	0.055	CDC45	cell division cycle 45
-3.0	0.047	0.093	KIAA0101	KIAA0101
-3.0	0.014	0.046	GPRC5A	G protein-coupled receptor, class C, group 5, member A
-3.0	0.008	0.035	MAD2L1	MAD2 mitotic arrest deficient-like 1 (yeast)

-2.9	0.034	0.076	SKA3	spindle and kinetochore associated complex subunit 3
-2.9	0.050	0.097	SHCBP1	SHC SH2-domain binding protein 1
-2.9	0.016	0.049	TRIP13	thyroid hormone receptor interactor 13
-2.8	0.028	0.068	CENPN	centromere protein N
-2.8	0.013	0.044	HIST1H3J	histone cluster 1, H3j
-2.8	0.050	0.096	CDCA3	cell division cycle associated 3
-2.8	0.013	0.044	TMPO	thymopoietin
-2.8	0.015	0.047	HIST1H4C	histone cluster 1, H4c
-2.7	0.022	0.059	POSTN	periostin, osteoblast specific factor
-2.7	0.004	0.029	HIST1H3F	histone cluster 1, H3f
-2.7	0.001	0.019	CD97	CD97 molecule
-2.7	0.005	0.031	CD9	CD9 molecule
-2.7	0.004	0.029	FABP5	fatty acid binding protein 5 (psoriasis-associated)
-2.7	0.001	0.019	SVIP	small VCP/p97-interacting protein
-2.7	0.003	0.027	TM4SF1	transmembrane 4 L six family member 1
-2.7	0.021	0.057	DIAPH3	diaphanous-related formin 3
-2.6	0.036	0.078	SKA2	spindle and kinetochore associated complex subunit 2
-2.6	0.024	0.062	NCAPH	non-SMC condensin I complex, subunit H
-2.6	0.005	0.030	SMAD3	SMAD family member 3
-2.6	0.036	0.078	FBXO5	F-box protein 5
-2.6	0.000	0.016	PRSS2	protease, serine, 2 (trypsin 2)
-2.5	0.003	0.027	ALDH1A3	aldehyde dehydrogenase 1 family, member A3
-2.5	0.048	0.095	FOXM1	forkhead box M1
-2.5	0.036	0.079	ECT2	epithelial cell transforming 2
-2.5	0.027	0.066	WDR76	WD repeat domain 76
-2.5	0.029	0.068	FAM129A	family with sequence similarity 129, member A
-2.5	0.003	0.028	KRT18	keratin 18
-2.5	0.039	0.083	LXN	latexin
-2.5	0.027	0.066	EMP2	epithelial membrane protein 2
-2.4	0.004	0.029	HLA-DQA2	major histocompatibility complex, class II, DQ alpha 2
-2.4	0.006	0.033	DHFR	dihydrofolate reductase
-2.4	0.002	0.027	PRUNE2	prune homolog 2 (Drosophila)
-2.4	0.029	0.068	KIAA1524	KIAA1524
-2.4	0.035	0.078	CFB	complement factor B
-2.4	0.033	0.074	CDCA8	cell division cycle associated 8
-2.4	0.002	0.022	KRT18	keratin 18
-2.3	0.014	0.047	APOBEC3B	apolipoprotein B mRNA editing enzyme, catalytic polypeptide-like 3B
-2.3	0.010	0.039	RPL22L1	ribosomal protein L22-like 1
-2.3	0.023	0.060	ZNF724P	zinc finger protein 724, pseudogene
-2.3	0.004	0.029	CDK2	cyclin-dependent kinase 2
-2.3	0.008	0.035	GPR1	G protein-coupled receptor 1
-2.3	0.035	0.078	SGOL2	shugoshin-like 2 (S. pombe)

-2.3	0.024	0.062	MCM6	minichromosome maintenance complex component 6
-2.3	0.002	0.026	HIST2H2BF	histone cluster 2, H2bf
-2.3	0.004	0.029	PRKCDBP	protein kinase C, delta binding protein
-2.3	0.030	0.071	ATAD2	ATPase family, AAA domain containing 2
-2.3	0.012	0.044	UBALD2	UBA-like domain containing 2
-2.2	0.002	0.023	TCF19	transcription factor 19
-2.2	0.040	0.084	ZWILCH	zwilch kinetochore protein
-2.2	0.000	0.016	HAUS8	HAUS augmin-like complex, subunit 8
-2.2	0.000	0.014	SH2D4A	SH2 domain containing 4A
-2.2	0.005	0.032	TEK	TEK tyrosine kinase, endothelial
-2.2	0.048	0.095	CENPA	centromere protein A
-2.2	0.007	0.034	EIF5AL1	eukaryotic translation initiation factor 5A-like 1
-2.2	0.005	0.032	NTM	neurotrimin
-2.1	0.000	0.014	ARHGAP29	Rho GTPase activating protein 29
-2.1	0.005	0.032	CLSPN	claspin
-2.1	0.046	0.092	RNASEH2A	ribonuclease H2, subunit A
-2.1	0.029	0.068	**CD200**	CD200 molecule
-2.1	0.015	0.049	UBE2S	ubiquitin-conjugating enzyme E2S
-2.1	0.027	0.066	HIST1H2AI	histone cluster 1, H2ai
-2.1	0.008	0.035	C1orf21	chromosome 1 open reading frame 21
-2.1	0.008	0.035	AURKB	aurora kinase B
-2.1	0.017	0.051	**CNN1**	calponin 1, basic, smooth muscle
-2.1	0.001	0.020	ASS1	argininosuccinate synthase 1
-2.1	0.028	0.067	EIF5AL1	eukaryotic translation initiation factor 5A-like 1
-2.0	0.006	0.033	EBF3	early B-cell factor 3
-2.0	0.024	0.061	**MYOCD**	myocardin
-2.0	0.004	0.029	SLC43A3	solute carrier family 43, member 3
-2.0	0.004	0.029	POLR2A	polymerase (RNA) II (DNA directed) polypeptide A, 220kDa
-2.0	0.001	0.019	PLA2G16	phospholipase A2, group XVI

Genes that were previously upregulated in cHL-derived fibroblasts are marked in bold.

Table S4. Genes upregulated after 48 hrs coculture of L-428 cells with NS cHL-derived fibroblasts compared with freshly mixed L-428 cells and NS cHL-derived fibroblasts.

Fold Change coculture/mock	p-value	False discovery rate (FDR)	Gene Symbol	Gene Description
9.1	0.000	0.000	IL1B	interleukin 1, beta
5.4	0.001	0.002	CCL26	chemokine (C-C motif) ligand 26
5.2	0.000	0.001	FDCSP	follicular dendritic cell secreted protein
4.5	0.000	0.000	TXNIP	thioredoxin interacting protein
4.4	0.000	0.001	IL2RA	interleukin 2 receptor, alpha
4.0	0.000	0.001	JUNB	jun B proto-oncogene
3.8	0.000	0.001	IFI27	interferon, alpha-inducible protein 27
3.6	0.000	0.000	IL6	interleukin 6
3.6	0.000	0.000	IDH2	isocitrate dehydrogenase 2 (NADP+), mitochondrial

3.5	0.001	0.002	CXCL8	chemokine (C-X-C motif) ligand 8
3.5	0.000	0.001	VCAM1	vascular cell adhesion molecule 1
3.3	0.000	0.001	EFEMP1	EGF containing fibulin-like extracellular matrix protein 1
3.3	0.000	0.001	SERPING1	serpin peptidase inhibitor, clade G (C1 inhibitor), member 1
3.2	0.000	0.001	TVP23A	trans-golgi network vesicle protein 23 homolog A (S. cerevisiae)
3.2	0.000	0.001	HCST	hematopoietic cell signal transducer
2.9	0.000	0.001	HMGCS1	3-hydroxy-3-methylglutaryl-CoA synthase 1 (soluble)
2.9	0.000	0.001	TGM2	transglutaminase 2
2.9	0.000	0.001	CFB	complement factor B
2.8	0.014	0.015	RPPH1	ribonuclease P RNA component H1
2.8	0.000	0.000	INHBE	inhibin, beta E
2.8	0.000	0.001	ASS1	argininosuccinate synthase 1
2.7	0.000	0.001	ANG	angiogenin, ribonuclease, RNase A family, 5
2.7	0.000	0.000	RRAGD	Ras-related GTP binding D
2.5	0.000	0.001	GLA	galactosidase, alpha
2.5	0.000	0.000	ALG13	ALG13, UDP-N-acetylglucosaminyltransferase subunit
2.4	0.000	0.001	PRAC1	prostate cancer susceptibility candidate 1
2.4	0.000	0.000	SOD2	superoxide dismutase 2, mitochondrial
2.4	0.000	0.000	BEX5	brain expressed, X-linked 5
2.4	0.001	0.002	SNORA5A	small nucleolar RNA, H/ACA box 5A
2.4	0.000	0.001	FUCA1	fucosidase, alpha-L- 1, tissue
2.3	0.000	0.000	C1S	complement component 1, s subcomponent
2.3	0.000	0.001	TNFRSF9	tumor necrosis factor receptor superfamily, member 9
2.3	0.000	0.001	DHRS3	dehydrogenase/reductase (SDR family) member 3
2.3	0.000	0.000	MID2	midline 2
2.3	0.001	0.002	KRT23	keratin 23 (histone deacetylase inducible)
2.3	0.002	0.003	IFITM1	interferon induced transmembrane protein 1
2.3	0.001	0.002	CD68	CD68 molecule
2.2	0.000	0.001	G0S2	G0/G1 switch 2
2.2	0.000	0.000	STAT5B	signal transducer and activator of transcription 5B
2.2	0.000	0.000	NIPSNAP1	nipsnap homolog 1 (C. elegans)
2.2	0.001	0.002	HSD17B8	hydroxysteroid (17-beta) dehydrogenase 8
2.2	0.001	0.001	MME	membrane metallo-endopeptidase
2.2	0.000	0.001	UBE2S	ubiquitin-conjugating enzyme E2S
2.2	0.000	0.000	ARMCX2	armadillo repeat containing, X-linked 2
2.2	0.000	0.001	IFI6	interferon, alpha-inducible protein 6
2.2	0.000	0.001	SLC50A1	solute carrier family 50 (sugar efflux transporter), member 1
2.2	0.001	0.001	IL32	interleukin 32

2.2	0.000	0.000	SLC26A4	solute carrier family 26 (anion exchanger), member 4
2.2	0.000	0.000	CYP1B1	cytochrome P450, family 1, subfamily B, polypeptide 1
2.1	0.002	0.003	SAT1	spermidine/spermine N1-acetyltransferase 1
2.1	0.000	0.001	WIPI1	WD repeat domain, phosphoinositide interacting 1
2.1	0.000	0.000	C4B	complement component 4B (Chido blood group)
2.1	0.000	0.001	BLVRB	biliverdin reductase B (flavin reductase (NADPH))
2.1	0.006	0.007	TFPI2	tissue factor pathway inhibitor 2
2.1	0.007	0.008	SELM	selenoprotein M
2.1	0.000	0.000	TDRKH	tudor and KH domain containing
2.1	0.000	0.001	CD80	CD80 molecule
2.1	0.002	0.003	C16orf95	chromosome 16 open reading frame 95
2.0	0.001	0.002	SIGLECL1	SIGLEC family like 1
2.0	0.001	0.002	BAIAP2L1	BAI1-associated protein 2-like 1
2.0	0.000	0.001	HIST1H2BK	histone cluster 1, H2bk
